# Effective Skin Rejuvenation by a Novel Antioxidant Biostimulating Treatment

**DOI:** 10.1111/jocd.70196

**Published:** 2025-04-25

**Authors:** Sofia Iglesia, Lily I. Jiang, Tatiana Kononov, Alisar S. Zahr

**Affiliations:** ^1^ Revision Skincare Irving Texas USA; ^2^ SGS North America, INC. Richardson Texas USA

**Keywords:** aesthetic dermatology, antioxidant, collagen, elastin, skin rejuvenation

## Abstract

**Background:**

Facial skin aging is a cumulative result of both intrinsic and extrinsic stressors. Chemical peels are commonly performed noninvasive skin rejuvenation procedures targeting these stressors. Innovation in chemical peels has remained limited, creating an opportunity for the industry to evolve with next‐generation technology.

**Aims:**

A novel topical antioxidant biostimulating treatment (ABT) was developed that utilizes a typical chemoexfoliation base as a vehicle to deliver a unique blend of antioxidant biostimulating acidic phytocompounds (ABAP). It was hypothesized that controlled delivery of ABAP could occur through a typical AHA/BHA chemical peel vehicle. These ABAPs, such as asiatic, ursolic, madecassic, and oleanolic acids, are known for skin rejuvenation in the papillary dermis.

**Patients/Methods:**

The ABT was tested in both ex vivo and in vivo settings to elucidate its mechanism of action and determine its efficacy and safety on 32 female subjects aged 38–60 years with Fitzpatrick skin types I–V.

**Results:**

The ABT upregulated elastin and collagen after superficial wounding of the skin by over 25% when compared to untreated or vehicle controls. Clinical evaluation by an expert grader demonstrated that the ABT significantly improved fine lines, wrinkles, tactile laxity, and overall appearance after three ABT sessions. Clinical photography demonstrated an improvement in fine lines, wrinkles, skin smoothness, laxity, radiance, and overall appearance.

**Conclusions:**

The ABT developed with ABAP technology was efficacious in improving facial skin aging for skin rejuvenation and safe for all skin tones.

## 1 Introduction

Chemical peels have climbed in popularity, ranking as the third most commonly performed noninvasive skin rejuvenation aesthetic procedure in the United States since 2021 [1, 2]. Chemical peels deploy caustic agents for controlled kerato‐coagulation and protein denaturation in the epidermis and dermis [3, 4, 5, 6]. Depending on the strength of the caustic agents, chemical peels can penetrate to different depths of the skin [3, 5]. Superficial chemical peels typically reach up to the epidermal‐dermal junction and have the advantage of instant effects with little to no downtime. Most superficial chemical peels contain alpha‐hydroxy acid (AHA), such as glycolic acid, lactic acid, and mandelic acid, or beta‐hydroxy acid (BHA), such as salicylic acid [6, 7, 8]. While chemical peels remain a cornerstone in aesthetic care, their technology is dated, with limited innovation in recent years [9]. There is a clear need for more advanced innovations that surpass the capabilities of traditional chemoexfoliation.

Plant‐derived bioactive compounds have been used for centuries as therapies for diseases, beauty, and well‐being/longevity [10, 11, 12, 13, 14]. Research in the past decades has identified many phytocompounds that have antioxidant, anti‐inflammatory, antimicrobial, healing, cytoprotective, and anticarcinogenic activities [10, 11, 12, 13, 14]. For example, asiatic acid, ursolic acid, madecassic acid, and oleanolic acid have been shown to have anti‐oxidative and anti‐inflammatory activities, as well as stimulate collagen synthesis [15, 16, 17, 18, 19]. Betulinic acid, malic acid, and nordihydroguaiaretic acid have been shown to attenuate oxidative stress by scavenging free radicals and inhibit matrix metalloproteinases (MMPs) [20, 21, 22, 23]. Specifically, both ursolic acid and betulinic acid downregulate MMP‐9 involved in elastin degradation to promote elastin synthesis [24, 25]. Incorporation of phytocompounds into topical formulations has been challenging due to their poor solubility and limited permeability into the skin [10, 11, 12, 13].

We hypothesized that a topical treatment formulated with a typical chemexfoliation AHAs and BHAs would be an effective method to facilitate the delivery of the antioxidant biostimulating acidic phytocompounds (ABAP) into the skin. During the chemical peel treatment, fissures may form among the keratinocytes and between the epidermal basal cells [4, 5, 6, 26]. This would allow the delivery of larger size molecules like the ABAP into skin cells. To this end, an antioxidant biostimulating treatment (ABT) was formulated to combine a unique blend of ABAP, including asiatic, ursolic, madecassic, and oleanolic acid, with commonly used chemical peel acids, lactic, glycolic, and salicylic acid to induce epidermal exfoliation and stimulate dermal rejuvenation.

The objective of this clinical study was to evaluate the ABT in a series of three progressive sessions at 1‐month intervals on female participants with mild‐to‐moderate facial photodamage. We hypothesized that the ABT would be effective in skin rejuvenation and improve the appearance of facial wrinkles and skin aging signs.

## 2 Methods

### 2.1 Ex Vivo Experiment

The ABT was evaluated in two ex vivo studies using living human skin explants obtained from abdominoplasty.

The first ex vivo study was performed on human skin explants of a 45‐year‐old Caucasian with Fitzpatrick Skin Type III to compare the effect of ABT versus untreated conditions. The treatment group received topically applied ABT at 2 mg/cm^2^ on the surface of the explant on Days 0, 2, 5, 7, and 8. Explants' surfaces were wiped clean before each treatment application. The control tissues did not receive any treatment (untreated), except the renewal of the culture medium on Days 1, 2, 5, and 7. Each condition was performed on six replicates.

The second ex vivo study was performed on human skin explant of a 42‐year‐old Caucasian with Fitzpatrick Skin Type III to compare the effect of ABT versus a vehicle‐control formula. The vehicle‐control formula contains the same industry‐trusted acids found in the ABT, without the ABAP. The treatment group and vehicle‐control group were topically treated with 2 mg/cm [2] of ABT or vehicle‐control, respectively, on Days 0, 1, 4, 6, and 8. Explants' surfaces were wiped clean before each treatment application. Each condition was performed on six replicates.

The following methodologies were performed in both ex vivo studies. Tissues underwent histological processing, in which tissues were either fixed in buffered formalin or frozen at −80°C for 24 h. Tissues processed in formalin were then dehydrated and embedded using a Leica EG 1160 embedding station. Five (5)‐μm‐thick sections were mounted on Superfrost histological glass slides. The frozen samples were then cut into 7‐μm‐thick sections using a Leica CM 3050 cryostat and mounted on glass slides.

In the first study, Masson's trichrome staining, Goldner variant, was performed on Days 0, 6, and 9 and assessed by microscopy to determine cell viability of the epidermal and dermal structures. The Mackenzie test was performed on tissues on Days 0 and 9 to evaluate the exfoliative effect of the ABT. Frozen skin sections were treated with the Mackenzie solution (sodium hydroxide solution). The number of stratum corneum cell layers was determined by microscopy and assessed using cellSens scoring software (Olympus, Tokyo, Japan).

Elastin immunostaining (Study 1) and collagen I immunostaining (Studies 1 and 2) were performed on frozen skin sections using an anti‐elastin polyclonal antibody (Novotec), diluted at 1:200 in PBS BSA 0.3% Tween 20 at 0.05% (w/w), and a polyclonal anticollagen I antibody (Abcam, ab138492‐1001), diluted at 1:1600 in PBS BSA 0.3% Tween 20 at 0.05% (w/w). The secondary antibodies were coupled with AlexaFluor 488 (Lifetechnologies, California, USA), and the nuclei were counter‐stained using propidium iodide. Signal intensity was quantified using the cellSens software (Olympus, Tokyo, Japan).

The Student's *t*‐test statistical analysis was performed evaluating treated tissues compared to control (Study 1) and vehicle‐control (Study 2) at Day 9. Statistical significance was achieved at *p < 0.05 and **p < 0.01.

### 2.2 In Vivo Clinical Study

#### 2.2.1 Study Population

Informed consent was obtained from all participants at the beginning of the clinical study. The study was conducted in accordance with all applicable guidelines for the protection of human subjects for research as outlined in 21 CFR 50, the accepted standards for Good Clinical Practice. Females aged 35–60 years of all skin tones (Fitzpatrick skin types I–VI) with mild‐to‐moderate facial fine lines, wrinkles, and tactile laxity were recruited. Subjects with a history of facial Herpes simplex, having used prescription retinoid products topically within the past 3 months or systemically within the past 12 months, over‐the‐counter retinol‐containing, anti‐wrinkle, skin‐lightening, or other topical or systemic products within 4 weeks were excluded from study enrollment. Subjects who were pregnant, breastfeeding, or planning a pregnancy during the study were also excluded from participation. A total of 36 subjects were enrolled in the study.

#### 2.2.2 Study Design

A single‐center, 12‐week clinical study was IRB‐approved and conducted from January to May 2022. This clinical study evaluated the efficacy and tolerability of an ABT when applied by a licensed esthetician in a series of three progressive sessions at 1‐month intervals. A Biocellulose Mask (BM) was additionally evaluated by the subjects for its soothing effect immediately post‐ABT application.

Qualified subjects were enrolled and underwent a 1‐week washout period prior to the baseline visit as described in Table 1. A series of three progressive ABT sessions was performed by a licensed esthetician in clinic at 1‐month intervals at baseline, Week 4, and Week 8 following the treatment protocol. Following baseline and for the duration of the 12‐week study, subjects were instructed to refrain from using any topical skincare products outside of what was provided and instructed to follow a skincare regimen.

**TABLE 1:** Skincare regimen utilized to support clinical study.

Washout period skincare regimen (1 week)	
Morning (AM)	Gentle foaming cleanser, basic facial moisturizer, basic sunscreen SPF 30
Evening (PM)	Gentle foaming cleanser, basic facial moisturizer

Smart antioxidant biostimulating treatment	
Steps	Instructions
Cleansing	Double cleansed the subjects global face with a Cleansing Gel
Prepping solution	Apply a defatting skin solution to the entire face.
Occlusive barrier	Applied an occlusive barrier to the mucus membranes
Antioxidant biostimulating treatment (ABT)	Apply the ABT to the entire face at 2‐min intervals At the final 2‐min interval, remove ABT with cool water‐soaked cotton
Biocellulose mask (BM)	Apply a soothing biocellulose mask to the entire face for 5–10 min
Post‐treatment serum	Apply the post‐treatment serum to the entire face

Skincare regimen (12 weeks)			
	Day of ABT (Baseline, Weeks 4 and 8)	Day 1 and 2 after ABT	Starting Day 3 after ABT (until next visit)
Morning (AM)	Gentle foaming cleanser post treatment serum basic sunscreen SPF 30	Gentle foaming cleanser post treatment serum basic sunscreen SPF 30	Gentle foaming cleanser basic facial moisturizer basic sunscreen SPF 30
Evening (PM)	Gentle foaming cleanser post‐treatment serum	Gentle foaming cleanser Post‐treatment serum	Gentle foaming cleanser basic facial moisturizer

At baseline, Weeks 4, 8, and 12 visits, subjects participated in clinical evaluation procedures, bioinstrument measurements, digital imaging, and completed self‐assessment questionnaires.

#### 2.2.3 Clinical Evaluations

Clinical grading of efficacy parameters was performed by expert graders at baseline (before and after treatment), Weeks 4 and 8 before treatment, and at Week 12. Efficacy parameters include tactile laxity, radiance, wrinkles, overall appearance of skin condition (healthy), visual skin smoothness, and fine lines using a modified Griffith's 10‐point scale, where a score of 0 = none, 1–3 = mild, 4–6 = moderate, and 7–9 = severe [27]. Half‐points were acceptable as necessary to more accurately describe the skin condition. Local cutaneous tolerability was evaluated by an expert grader by assessing the signs of erythema and dryness, and by subject reporting the degree of burning, stinging, and itching of the global face using a 4‐point scale where a score of 0 = none, 1 = mild, 2 = moderate, and 3 = severe.

#### 2.2.4 Bioinstrumentation and Clinical Photography

The Tewameter TM300 (Courage + Khazaka electronic GmbH, Köln, Germany) was utilized to measure the passive transfer of water through the stratum corneum (transepidermal water loss [TEWL]) at baseline, Week 4 and Week 8 before the chemical treatment and at Week 12. Tewameter measurement was taken on the center of each subject's right or left cheek per randomization.

Facial clinical photography of the subject's global face (right, center, and left) was taken at baseline, Weeks 4, 8, and 12 using the VISIA‐CR photostation (Canfield Imaging Systems, New Jersey, USA) with a canon Mark II digital SLR camera (canon incorporated, Tokyo, Japan) under standard light, raking light, and polarized light conditions. Standard 2 images were used to analyze wrinkles at the forehead and nasolabial fold areas using Vaestro software (Canfield, New Jersey, USA). Standard images were also used to generate 3D skin roughness images using ImagePro Plus software (Media Cybernetics, Maryland, USA).

#### 2.2.5 Subject Self‐Assessment Questionnaire

Subjects completed self‐assessment questionnaires where they rated statements regarding skin condition on a 5‐point scale where a score of 1 = worst condition/completely disagree to 5 = best condition/completely agree. Additionally, at Week 12, subjects were asked to indicate whether they had experienced any skin peeling/flaking in the days following each treatment.

#### 2.2.6 Statistical Analysis

Statistical analysis was performed on the intent‐to‐treat population, which included all subjects who received the ABT treatment and participated in at least one postbaseline evaluation. All statistical tests were two‐sided at a significance level of α = 0.05.

Mean change from baseline was calculated for postbaseline time points. The Wilcoxon signed‐rank test was performed to determine the significance of changes from baseline for clinical evaluation and tolerability parameters. A decrease in score indicates an improvement for the indicated clinical efficacy parameter and tolerability/safety for the test material.

Paired *t*‐test analysis was conducted for Tewameter and image analysis data. A decrease in Tewameter values reflects an improvement in the barrier properties of the skin. The null hypothesis was that the mean change from baseline is zero.

Self‐assessment questionnaire responses were analyzed using top box analysis. Percent subject agreement was determined for each parameter, and subject satisfaction was considered when subjects scored parameters from 4 (slightly agree) to 5 (completely agree) at Week 8 and 12. A higher percentage of favorable responses with a significant *p* value indicates positive subject perception of the test material.

## 3 Results

### 3.1 Ex Vivo Experiment

**FIGURE 1:**
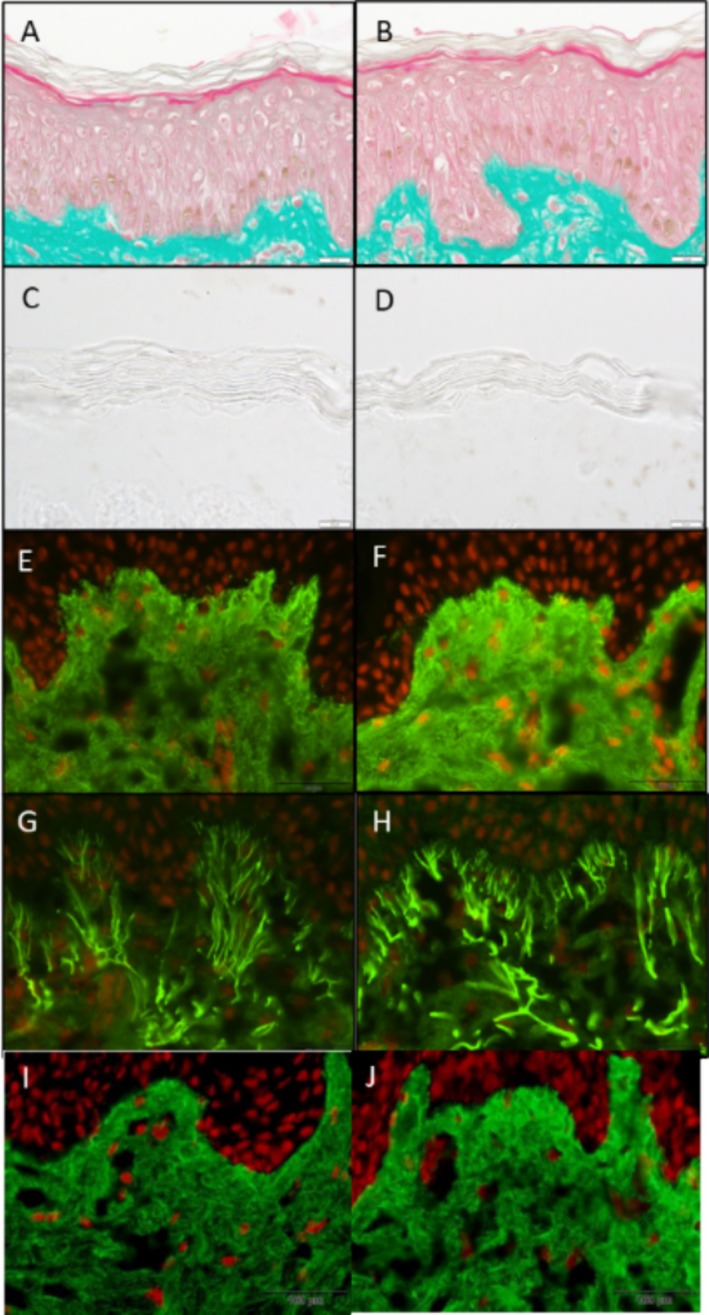
Ex vivo Study 1 evaluating the ABT in Masson's trichrome staining, Goldner variant to determine cell viability of the epidermal and dermal structures on (A) nontreated control, and (B) treated tissues at Day 9. Scale bar = 20 μm. Mackenzie test was implemented to determine the number of corneocyte layers and stratum corneum thickness on (C) non‐treated control, and (D) treated tissues at Day 9. Scale bar = 20 μm. Immunostaining of collagen I (E, F) and elastin (G, H) of the papillary dermis (green) and nuclei (red) on (E, G) nontreated control tissues, and (F, H) treated tissues at Day 9. Scale bar = 50 μm. Ex vivo Study 2 (I‐J) evaluating the ABT under immunostaining of collagen I of the papillary dermis (green) and nuclei (red) on (I) vehicle control–treated tissues, and (J) ABT‐treated tissues at Day 9. Scale bar = 100 μm.

Masson trichome stain Goldner variant of the epidermal and dermal layers showed the ABT treatment was not detrimental to cell viability. Additionally, an increase in stratum corneum lamination on Day 6 and a decrease in stratum corneum lamination and thickness on Day 9 were observed. The Mackenzie test further corroborates and quantifies these findings, demonstrating that the number of corneocyte layers decreased by 14% at Day 9 compared with control (**p < 0.01), and the stratum corneum thickness decreased by 14% at Day 9 compared with the control (*p < 0.05) (Figure 1A–D).

The ABT increased collagen I expression by 29% (*p < 0.05) when compared to the untreated control (Figure 1E,F) and by 26% (**p < 0.01) when compared to the vehicle control in the papillary dermis (Figure 1I,J). Furthermore, ABT increased elastin expression by 37% (**p < 0.01) when compared to the untreated control in the papillary dermis (Figure 1G,H).

### 3.2 In Vivo Clinical Study

A single‐center, open‐label, IRB‐approved, 12‐week clinical study was carried out to evaluate the efficacy and tolerability of the ABT. Thirty‐two [28] females, aged 35–60, including Fitzpatrick skin types I–V, with mild‐to‐moderate facial photoaging completed the clinical study (Table 2). Two [2] subjects were lost to follow‐up and two subjects requested to be withdrawn. Subject disposition and demographic information of the study population (Supplementary Table S1).

**TABLE 2:** Clinical study demographics of completed subjects.

**Demographics**		
Sample Size	32	
**Age**		
Average	53	
Min	38	
Median	55	
Max	60	
**Ethnicity**		
	***N***	**%**
Black	5	16%
Caucasian	24	75%
Asian	1	3%
Hispanic	2	6%
**Fitzpatrick skin type**		
	***N***	**%**
I	2	6%
II	16	50%
III	7	22%
IV	4	13%
V	3	9%

**FIGURE 2:**
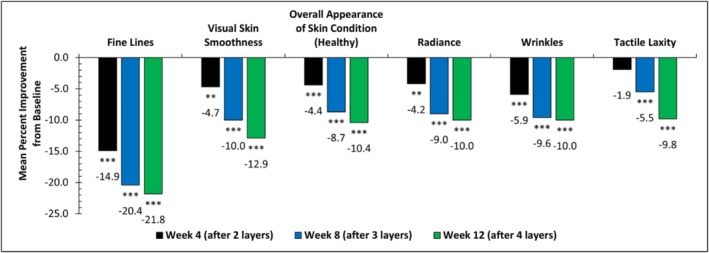
Mean percent improvement of clinical efficacy parameters across time points from baseline. Statistical significance was achieved at *p ≤ 0.05, **p ≤ 0.01, ***p ≤ 0.001.

Clinical evaluation of facial skin conditions showed that the ABT statistically significantly and progressively improved all evaluated facial aging signs, including fine lines, visual skin smoothness, overall appearance of skin condition (healthy), wrinkles, radiance, and tactile laxity (Figure 2). Specifically, a 14.9% mean improvement in fine lines was achieved at Week 4 (after one treatment), a 20.4% mean improvement at Week 8 (after two treatments), and a 21.8% mean improvement at Week 12 (after three treatments) (***p < 0.001) compared to baseline. At Week 12, 80% of subjects demonstrated improvement in skin laxity with a 9.8% mean improvement compared to baseline, and over 80% of subjects showed improvement in overall appearance of skin condition.

**FIGURE 3:**
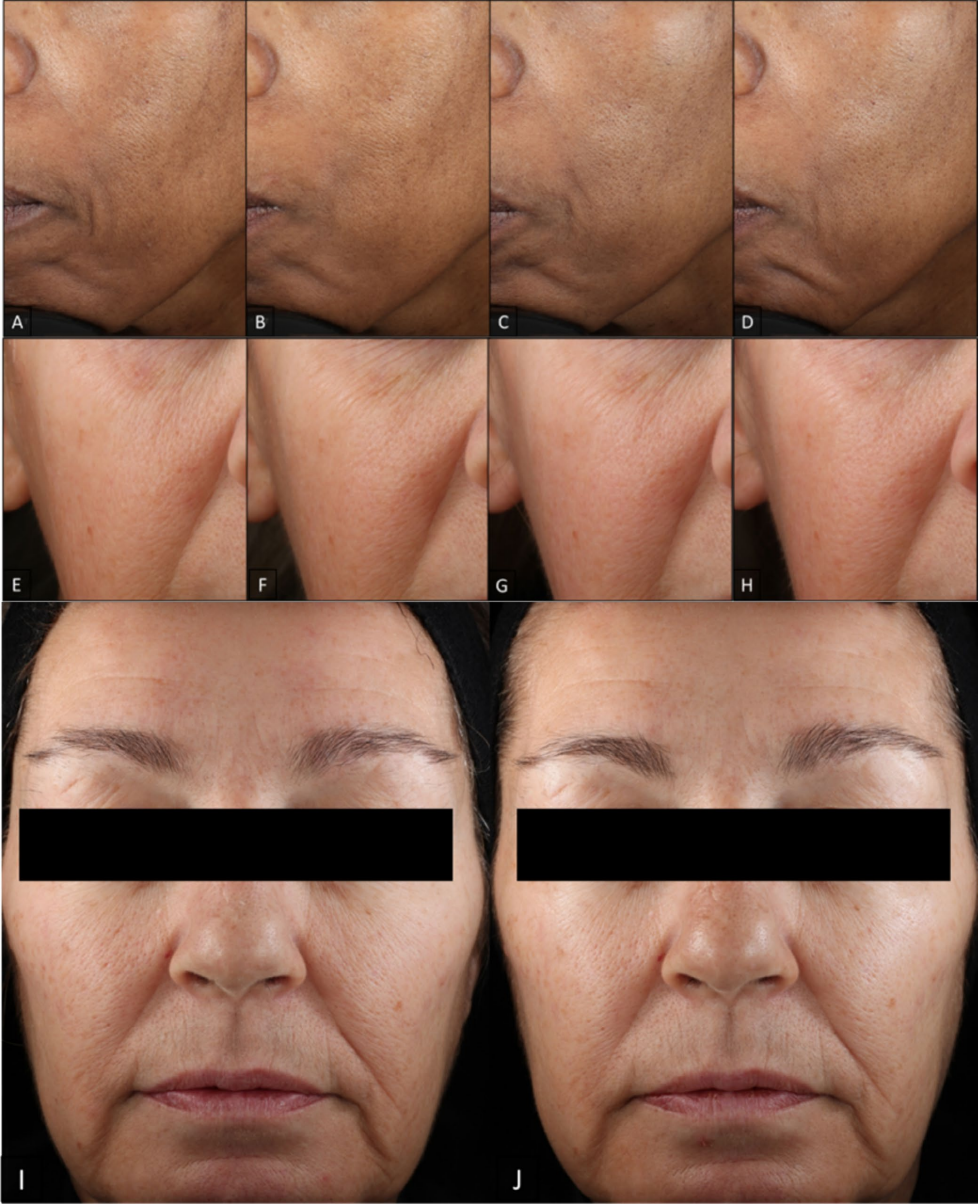
VISIA‐CR clinical photography of female subjects age 49 with Fitzpatrick skin type V left cheek in standard light (A–D); age 57, Fitzpatrick skin type III right cheek in raking light (E–H); and age 59, Fitzpatrick skin type III in raking light (I, J) at Baseline (A, E, I), Week 4, after 2 ABT layers (B, F), Week 8, after 3 ABT layers (C, G), and Week 12, after 4 ABT layers (D, H, J).

**FIGURE 4:**
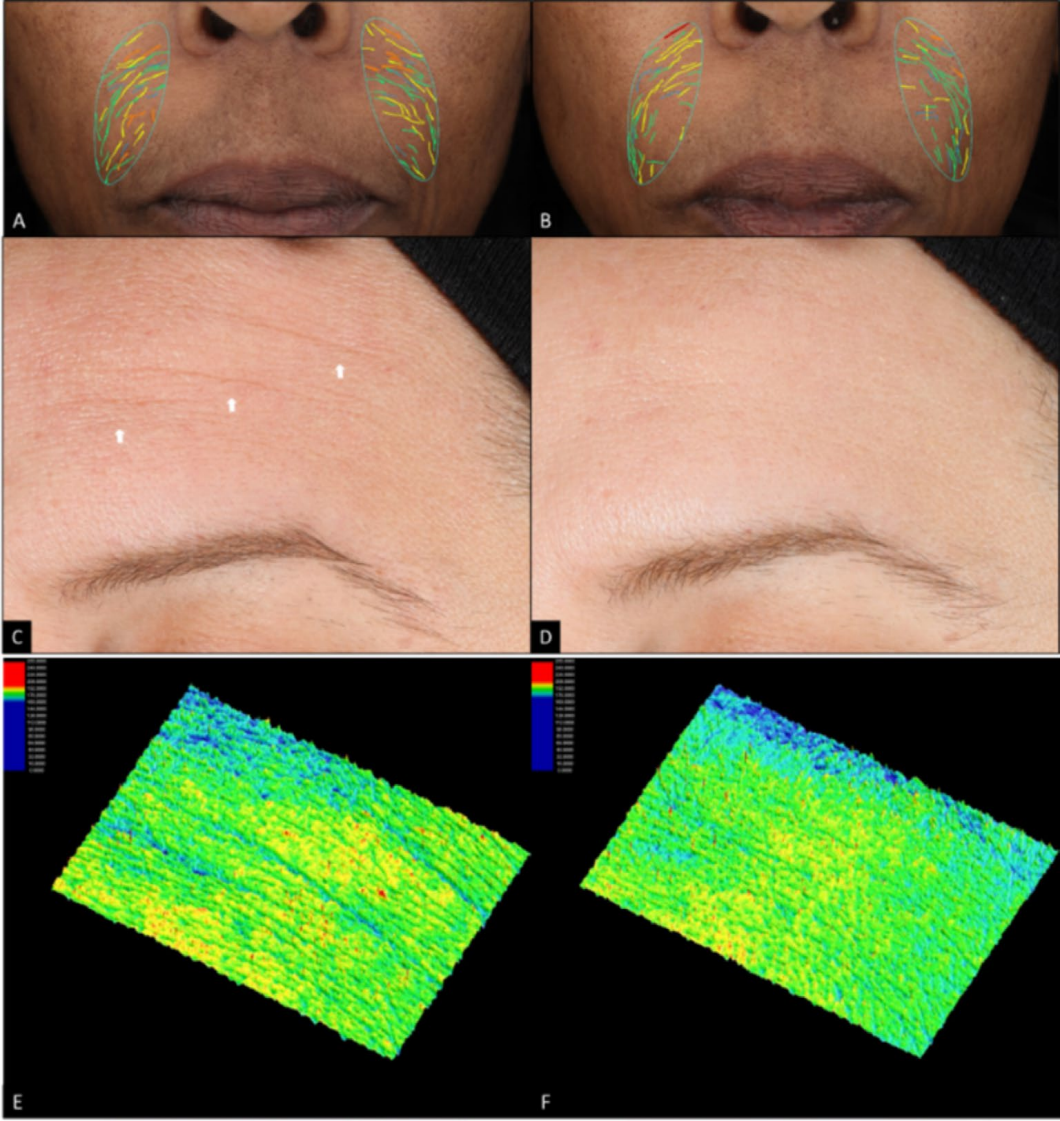
VISIA‐CR Clinical Photography in raking light with Vaestro nasolabial folds analysis of a female subject age 59, Fitzpatrick skin type V (A, B) and in standard light with 3D roughness analysis of a female subject age 43, Fitzpatrick skin type III (C–F). Baseline (A, C, E), and Week 12, after 4 ABT layers (B, D, F).

Visual improvements in fine lines, wrinkles, skin smoothness, laxity, radiance, and overall appearance are seen across varying Fitzpatrick skin types, and continued improvement is seen when subjects continue with the progressive ABT series. The improvement of facial skin conditions was well captured in clinical digital imaging. Photographs of a subject age 49 with Fitzpatrick skin type V clearly showed progressive improvement in fine lines and skin smoothness at each time point (Figure 3A–D). Another subject age 59 with Fitzpatrick skin type III showed progressive improvement in wrinkles and radiance (Figure 3E–H) as well as laxity and overall healthy skin appearance (Figure 3I,J). Improvement in forehead and nasolabial fold fine lines and wrinkles is visually represented in Figure 4.

The ABT not only demonstrated efficacy in improving wrinkles and fine lines, but also in skin roughness. A female subject age 43 with Fitzpatrick skin type III demonstrated visual improvements in forehead fine lines and wrinkles in standard light at Week 12 compared to baseline (Figure 4C,D). Further 3D skin roughness imaging analysis demonstrated additional improvement in skin roughness on the same subject at Week 12 compared to baseline (Figure 4E,F).

Importantly, the majority of the participants noticed the improvement in their skin quality and reported favorably for the ABT series when compared to baseline. (***p < 0.001).

Week 4 after the first treatment with two ABT layers:

○ 77% of subjects favorably agreed that their appearance of pores had improved.
○ 71% of subjects favorably agreed that their skin is smooth.

Week 8 after second treatment with three ABT layers.

○ 88% of subjects favorably agreed that their skin clarity improved.
○ 85% of subjects favorably agreed that their skin tone evenness improved.
○ 82% of subjects favorably agreed that their wrinkles improved.

Week 12 after third treatment with four ABT layers:

○ 94% of subjects favorably agreed that their skin clarity improved.
○ 91% of subjects favorably agreed that the appearance of their pores improved.
○ 91 of subjects favorably agreed that their skin is smooth.
○ 88% of subjects favorably agreed that their skin texture and fine lines improved.

The ABT was well tolerated among subjects; no adverse event related to the ABT was reported throughout the 12‐week clinical study. Immediately after the first ABT procedure, there were no reports of dryness, burning, stinging, or itching. Subjects experienced mild erythema immediately after treatment, which statistically significantly reduced over time. Three [3] subjects reported mild dryness, and one [1] subject reported mild itching at Week 4, which resolved at Week 8. One [1] subject reported mild dryness at Weeks 4 and 8, which resolved by Week 12. Throughout the study, there were no reports of burning or stinging. The gentleness of the ABT experience could have been further aided by the application of the BM after the treatment. 97% of subjects favorably agreed that the BM provided immediate cooling relief and felt calming and soothing on the skin post‐ABT application (***p < 0.001).

The tolerability of the antioxidant‐biostimulating treatment was further proved by instrument measurement. Tewameter measurement, which measures the TEWL and reflects the integrity of the skin barrier, showed no statistically significant changes at any time points post‐treatment. The majority of the subjects (68.8%) did not experience skin peeling in the days following each treatment.

## 4 Discussion

Facial skin is the most apparent place to witness the aging process as it is at the forefront to endure both the extrinsic and intrinsic aging factors [29, 30, 31]. There has been an increasing need for safe, effective, and scientifically driven products and treatments to revert facial skin aging by aestheticians and their patients. We have developed the novel ABT that combines a blend of ABAP with commonly used chemical peel ingredients, alpha and beta hydroxy acids. The ABAP is unique in its composition and is specifically selected for its net antiaging effects, including upregulating collagen and elastin, downregulating MMPs, and quenching both nitrogen‐ and oxygen‐free radicals. To circumvent the difficulty of delivering large molecular size phytocompounds into the proper layers of the skin where they can fully exert their mechanism of action, they are combined with hydroxy acids to formulate the ABT. During the ABT procedure, the AHAs and BHA momentarily disrupt the skin barrier by creating epidermal fissures, allowing optimal delivery of the ABAP into the skin to promote collagen and elastin neosynthesis for enhanced dermal repair and skin regeneration.

Through ex vivo studies, the ABT demonstrated an increase in stratum corneum lamination compared to the control on Day 6. This indicates that the stratum corneum has been weakened due to the exfoliant activity of the AHAs and BHA in the ABT. Only tight junctions at the end of the corneocytes ensure their cohesion. On Day 9, the corneocyte layers previously weakened are detached from the horny layers, and consequently, a decrease in stratum corneum thickness and lamination is observed. Together with the 14% decrease in corneocyte layers and a 14% decrease in stratum corneum thickness, these results elucidate the mechanism of action of the industry‐trusted acids in the ABT in superficially wounding the skin for optimal controlled delivery of the ABAP. Furthermore, the ABT stimulated a statistically significant increase in collagen and elastin expression in the papillary dermis compared to the untreated control and the vehicle control consisting of the typical chemical acids. These results indicate that the ABAP optimally penetrated the papillary dermis to induce dermal rejuvenation. The increase in collagen and elastin expression can be attributed to the ABAP, including asiatic acid, ursolic acid, madecassic acid, and oleanolic acid, which have been shown to stimulate the expression of collagen and elastin in cultured fibroblast cells [15, 16, 17, 18, 19]. These findings highlight that the ABAP is next‐generation technology that surpasses the limitations of traditional chemical peel acids by stimulating direct dermal rejuvenation.

After establishing the mechanism of action of the ABT, the safety and efficacy of the ABT were evaluated in a clinical trial on healthy women with mild‐to‐moderate photodamage. The ABT was designed to be a series of three progressive treatments every four weeks. We reasoned that the addition of the ABAP can help enhance the net antiaging benefits of the treatment. Clinical grading of the ABT effect was done after 4 weeks of each treatment and right before the next treatment. Results from the clinical study showed progressive improvement at each time point for all clinically evaluated grading parameters, including fine lines, visual skin smoothness, overall appearance of skin condition (healthy), wrinkles, radiance, and laxity.

The ABT was tolerated by all participants that included all skin tones from light to dark. There were no significant changes in facial dryness scores and no significant changes in TEWL measurement. The majority of the participants reported not noticing any skin peeling/flakes during the course of the study. Maintaining skin barrier integrity is essential for skin health [28]. The ABT achieved its functional benefits to skin rejuvenation without damaging the skin barrier integrity. It is also worth noting that 11.1% and 8.3% of the participants in this clinical trial were dark skin tone with Fitzpatrick skin type IV and V, respectively. None experienced any issues with the ABT during the 12‐week period, and all showed visible improvement on facial skin aging parameters (Figure 3). This result further supports the safety of this product to all skin tone people inclusively.

Skin quality is an important factor to human attractiveness and has great social and psychological impact on people's daily life [31]. Recently published consensus results from a group of dermatologists point to four perceptual aspects that define skin quality: skin tone evenness, skin surface evenness (texture), skin firmness, and skin glow [32]. We have shown here that the uniquely formulated topical ABT is efficacious in improving skin quality in all four skin quality aspects, adding an attractive skin rejuvenation option for aesthetic practices/providers.

The results in this present study demonstrated to be well tolerated with minimal downtime post‐treatment for subjects with Fitzpatrick skin types I–V. Additionally, there were no reports of adverse events, including Postinflammatory hyperpigmentation (PIH) throughout the clinical study, indicating that patients susceptible to PIH may benefit from the ABT. Therefore, a future study will be performed to demonstrate the ABT in improving skin aging concerns and assisting in mitigating PIH in patients with Fitzpatrick skin types V–VI.

The clinical presentation here demonstrated improvement in skin quality with a basic skincare regimen without a vehicle‐control, thus a limitation of this study. Additionally, the inclusion of skin elasticity measurements such as the cutometer would have complemented the investigator results. Future research could consider incorporating both vehicle‐control groups and instrumental methods to enhance the assessment of skin quality and elasticity.

## Author Contributions

The study was sponsored by Revision Skincare. Dr. Lily I. Jiang is a consultant for Revision Skincare and was involved in the discussion of the clinical study design and image/data review. Ms. Iglesia and Dr. Zahr analyzed the images and statistical data. Both are employees of Revision Skincare. Ms. Kononov is a consultant for Revision Skincare and was involved in the design of the clinicals. All authors contributed to the draft and review of the manuscript.

## Ethics Statement

This study, C22‐D008, was approved by Advara Investigational Review and registered in ClinicalTrials.gov: NCT06629792. This study was conducted in accordance with all applicable guidelines for the protection of human subjects for research as outlined in the United States FDA 21 CFR Part 50, in accordance with the accepted standards for Good Clinical Practices (GCPs). Written informed consent and photography consent were obtained from all subjects prior to inclusion in the study.

## Conflicts of Interest

The study was sponsored by Revision Skincare. Dr. Lily I. Jiang is a consultant for Revision Skincare and was involved in the discussion of the clinical study design, and image/data review. Ms. Iglesia and Dr. Zahr analyzed the images and statistical data. Both are employees of Revision Skincare. Ms. Kononov is a consultant for Revision Skincare and was involved in the design of the clinicals. All authors contributed to the draft and review of the manuscript.

## Supporting information


**Table S1.** Subject deposition and summary of demographic information of the intended to treat population.

## Data Availability

The data that support the findings of this study are available from the corresponding author upon reasonable request.
