# Supplementary figures and images for: Effective Skin Rejuvenation by a Novel Antioxidant Biostimulating Treatment
Source: J Cosmet Dermatol. 2025 Apr 25;24(4):e70196. doi: 10.1111/jocd.70196 (PMC12023018; doi:10.1111/jocd.70196)

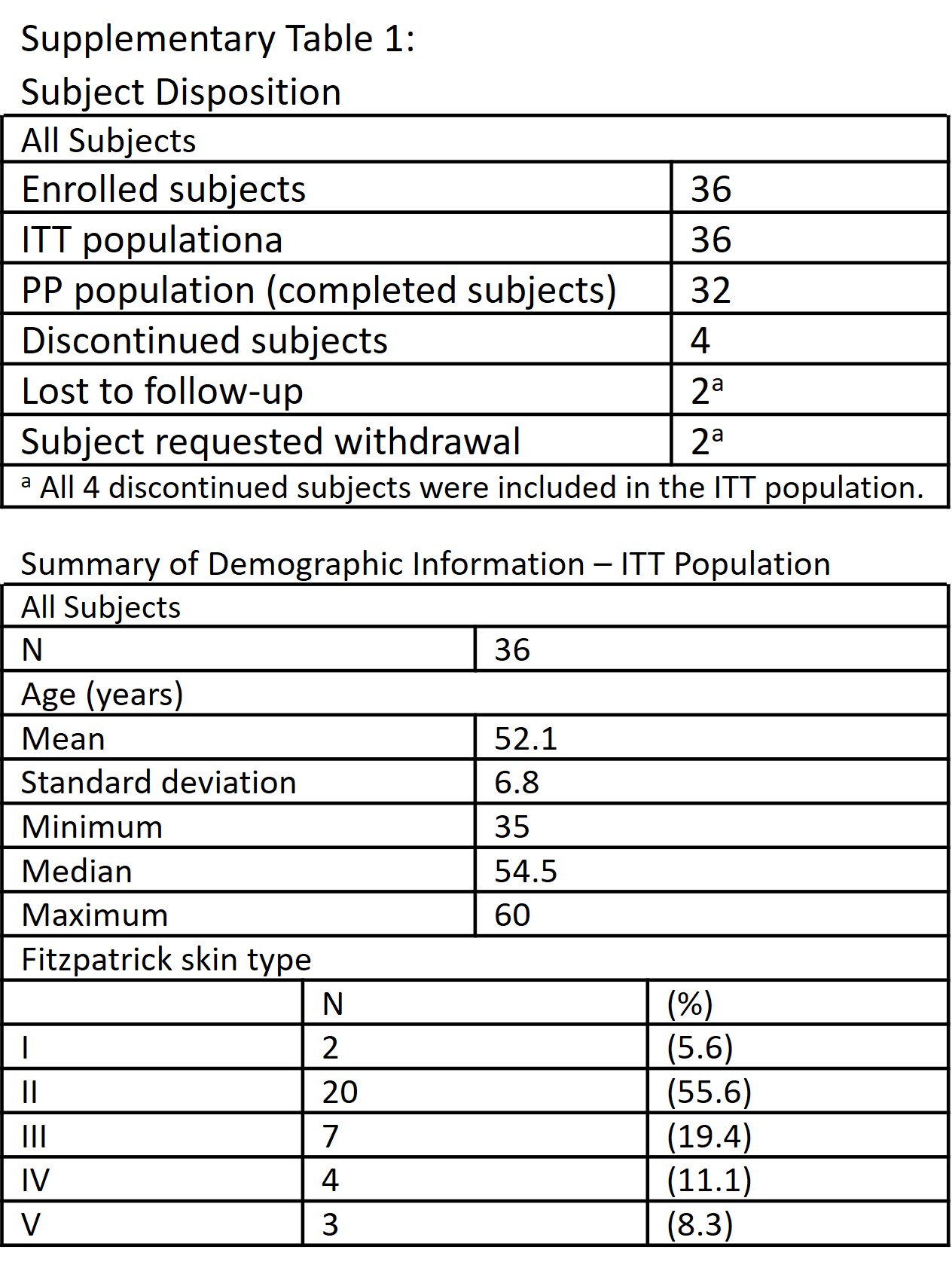

Supplement: Supplementary file 1 — Table S1. Subject deposition and summary of demographic information of the intended to treat population. [file JOCD-24-e70196-s001.jpg]
